# Physiotherapy Approach to a Stage V Parkinson’s Disease Patient: A Case Report

**DOI:** 10.7759/cureus.47549

**Published:** 2023-10-23

**Authors:** Shruti S Bhoge, Pallavi Harjpal, Swati Gupta

**Affiliations:** 1 Department of Neurophysiotherapy, Ravi Nair Physiotherapy College, Datta Meghe Institute of Higher Education and Research (DU), Wardha, IND

**Keywords:** case report, lsvt-big, lee silverman voice treatment, striatal hand deformity, physiotherapy, parkinson's disease

## Abstract

Parkinson’s disease (PD) is a neurodegenerative disorder caused due to decreased dopamine, a neurotransmitter, advancing to a range of motor and non-motor attributes. There is a death of dopamine-producing neurons (dopaminergic neurons) in the Substantia Nigra. Bradykinesia, postural instability, resting tremor, and rigidity are four main symptoms in this patient. A variety of other symptoms, like hypomimia, micrographia, freezing gait, decreased movement amplitude, constipation, cognitive impairments, etc., can be seen in this patient. In this paper, we report a 62-year-old female with stage 5 PD with chief complaints of uncoordinated movements, weakness, and difficulty in daily activities. She was treated with strengthening, stretching, Lee Silverman Voice Treatment (LSVT) BIG, bed mobility, gait training with auditory cueing, balance training, etc. LSVT-BIG enhances motor function by incorporating high amplitude motions of high intensity, consisting of numerous repetitions and progressive complexity. At the end of three weeks, the patient had improved strength, static and dynamic balance, gait, and quality of life.

## Introduction

Parkinson’s disease (PD), introduced by Dr. James Parkinson, is a gradual deterioration of nervous tissue over a long period, expressed in various motor and non-motor characteristics [1,2]. The apparent cause is the decreased dopamine (a neurotransmitter) release, courtesy of the loss of dopaminergic neurons in the substantia nigra region [3]. The four predominant features of PD are bradykinesia (slow/ sluggish movements), tremors (pin-rolling type), rigidity (cogwheel type,) and instability in posture [4]. In 2021, more than 42 cases per 100,000 people were prevalent in India and over ten million globally [5,6]. One study even indicated that the count of patients will only increase over the next few decades [7,8]. So, education, early treatment to reduce disability, and prevention are very pertinent.

Dopamine is a neurotransmitter created in Substantia nigra by the dopaminergic neuron. In PD, there is decreased production of this neurotransmitter, which leads to toxic excitation. Its primary function is inhibiting unnecessary movement. However, its deficiency and destruction of dopaminergic neurons give way to various motor, cognitive, affective, and sensory disturbances [9,10]. Apart from the cardinal features, hypomimia (face with no expression), micrographia, festinating or freezing gait, dystonia, anhedonia, constipation, mood changes, memory deficits, etc. are some other features that might be present [11,12].

Physiotherapy in PD focuses on enhancing the function of the upper and lower extremities, transfers, body posture, balance, etc., by using interventions like strengthening exercises, stretching, Lee Silverman Voice Treatment (LSVT) BIG, bed mobility exercises, and gait training with cueing and transfer techniques [13,14]. LSVT BIG is an intervention known to enhance motor function in PD patients. High-amplitude motions that are taught through several repetitions, high intensity, and gradually increasing complexity are the primary focus of LSVT-BIG.

This paper presents the case of a 62-year-old female patient with stage 5 (Hoehn and Yahr Scale) PD [15].

## Case presentation

A 62-year-old female diagnosed with Parkinsonism for 11 years came to the Neuro Outpatient Department (OPD) complaining of uncoordinated movements, restlessness, and weakness in the whole body. She is also a known case of hypertension 20 years back and hypothyroidism seven years back. The patient gave a history of discontinuing antihypertensives in the prior month. She also complained of shoulder joint pain and difficulty walking independently. She also suffered from constipation but had normal bladder habits and had no addictions.

Clinical findings

**Figure 1:**
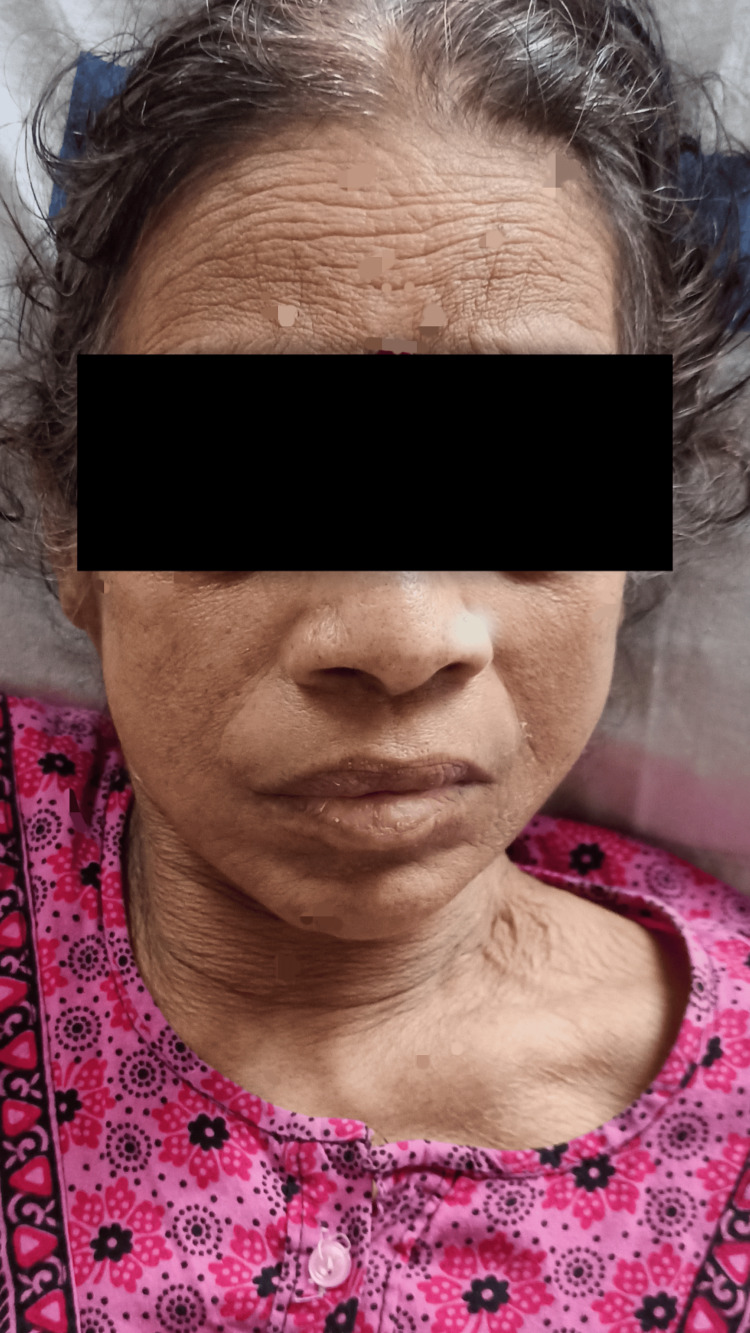
Mask-like face

**Figure 2:**
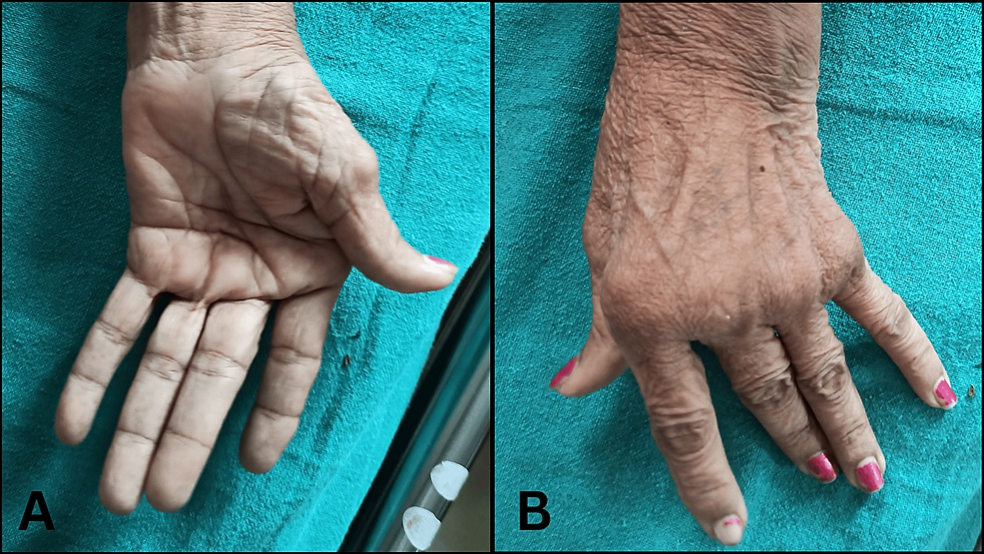
Striatal hand deformity A: Palmar aspect B: Dorsal aspect

On observation, the patient had a mesomorph body type. She was lying in the supine position and her vitals were stable. The patient had a mask-like face (Figure 1) and pill-rolling tremors. Her right hand was deviated medially (ulnar deviation), and there was tightness in finger flexors, known as striatal hand deformity, as shown in Figure 2. She needed mild assistance for sitting, but for standing and walking, she needed moderate aid. She had a festinating gait. Berg's balance score was 4, i.e., high risk of fall.

Tables 1, 2 show findings of muscle tone (Tone Grading System) of the upper extremities and reflexes of the patient.

**Table 1:** Muscle tone of upper extremities 1+: Decreased tone, 2+: Normal tone, 3+: Increased tone

Muscles	Right	Left
Shoulder		
Flexors	2+	2+
Extensors	2+	2+
Abductor	2+	2+
Adductor	2+	2+
Elbow		
Flexors	2+	2+
Extensors	2+	2+
Wrist		
Flexors	2+	3+
Extensors	2+	1+
Ulnar deviators	2+	3+
Radial deviator	2+	1+
Fingers		
Flexors	2+	3+
Extensors	2+	1+

**Table 2:** Reflexes +: Diminished reflex, ++: Normal reflex, +++: Exaggerated reflex

Reflexes	Right	Left
Biceps	++	++
Triceps	++	++
Knee	+++	++
Ankle	++	++
Plantar response	Extensor	Extensor

Investigations

The routine CBC (Complete Blood Count) test revealed that the patient was severely anemic, and the hemoglobin level was found to be 6.9 gm/dl (normal: 14-16gm/dL) as well as raised creatinine to 1.8 mg/dL (normal: 0.5-1.2 mg/dL) was also found in KFT (kidney function test) results.

Timeline

In August 2023, the patient was admitted to the Neurology ward, but the physiotherapy call was delayed due to high blood pressure. The patient was referred for physiotherapy, and after four days, the patient reported normal blood pressure. The patient received treatment for three weeks.

Therapeutic intervention

**Figure 3:**
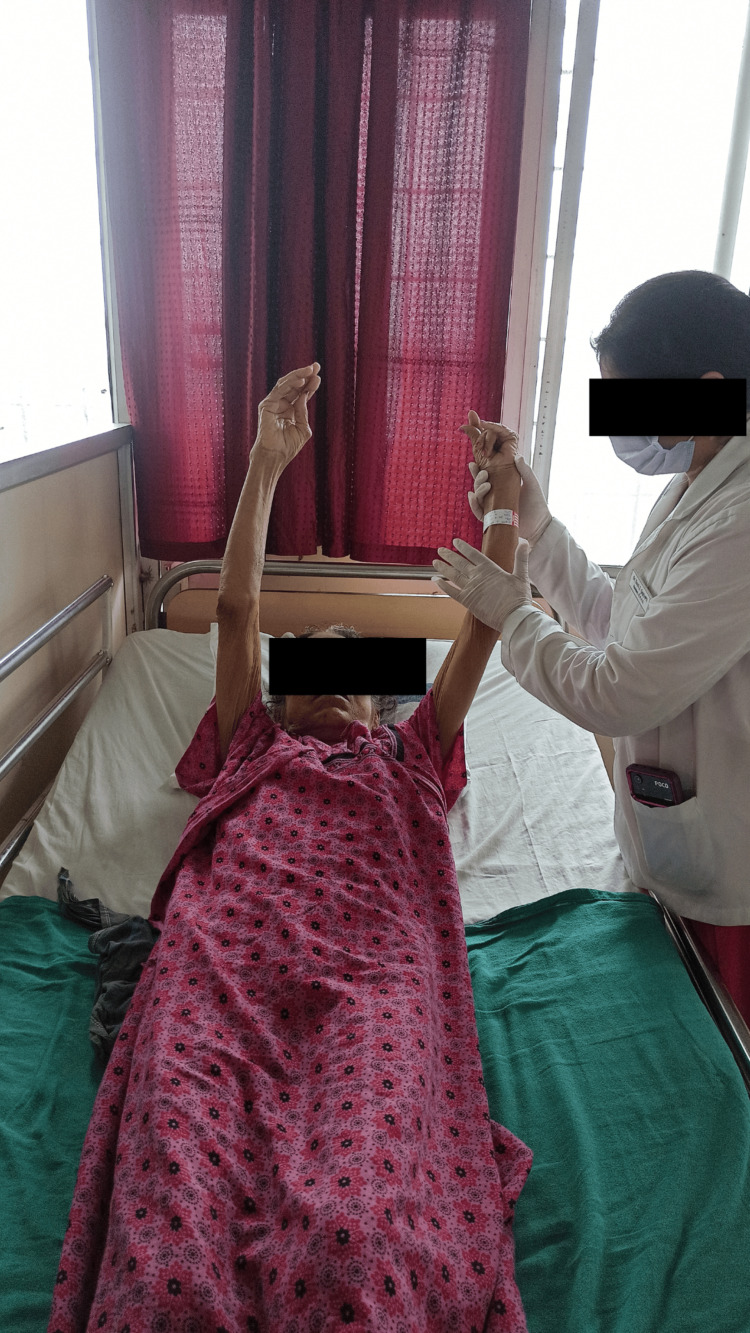
AROM exercises for upper limb AROM: Active range of motion

**Figure 4:**
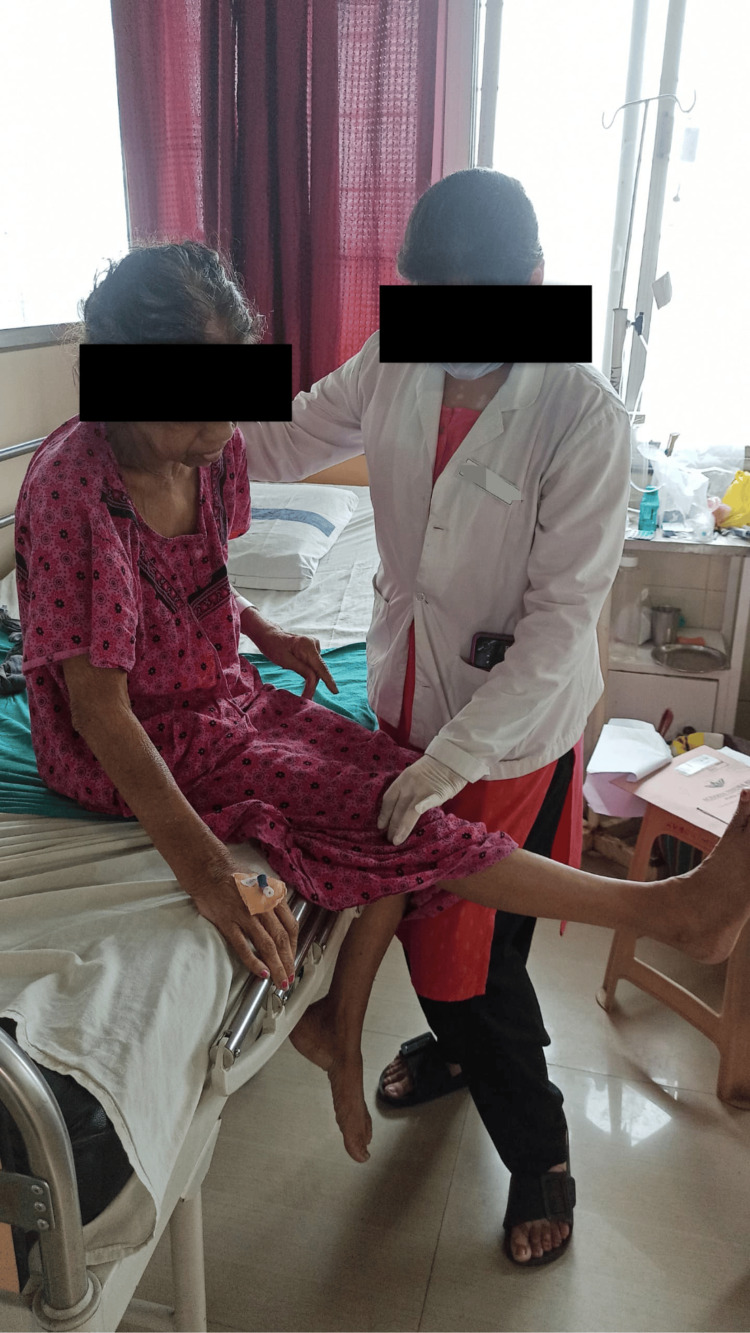
Strengthening for quadriceps muscle

**Figure 5:**
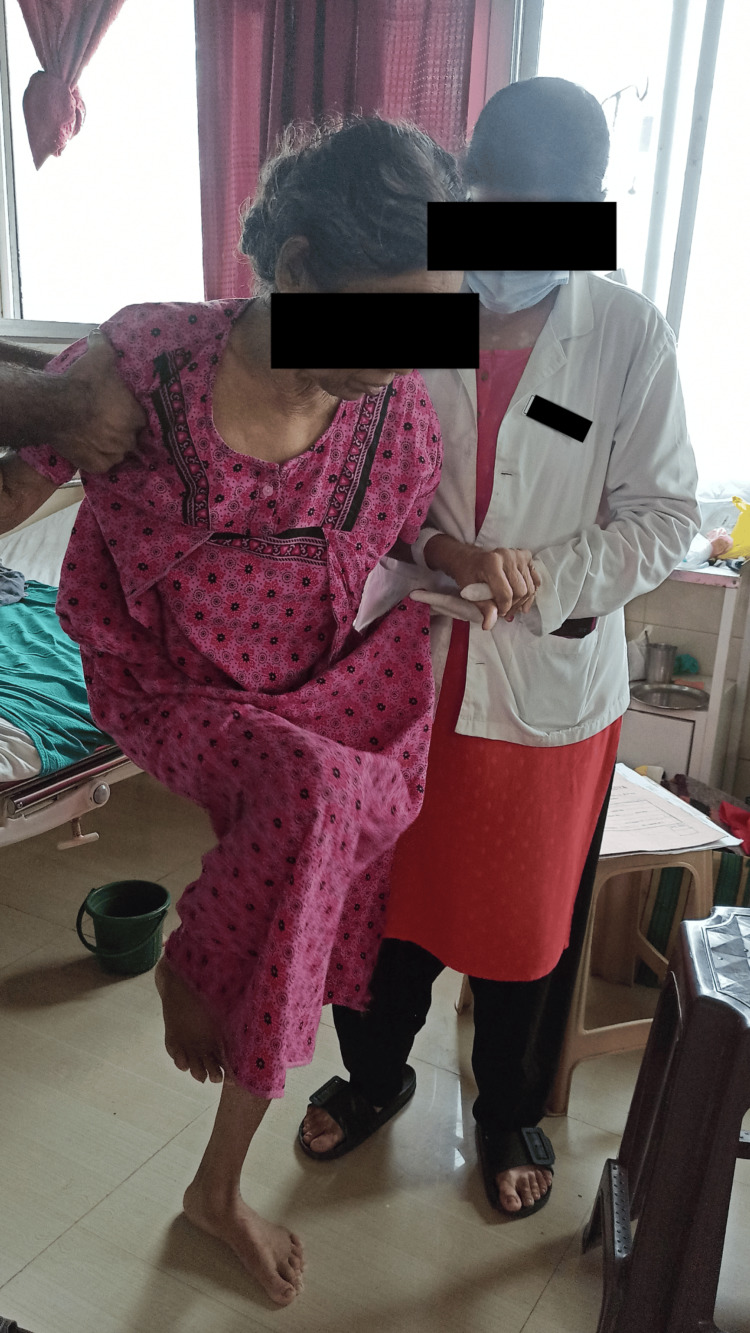
Spot marching

Table 3 and Figures 3-5 give a brief summary and show the physiotherapy intervention received by the patient over a span of three weeks, respectively.

**Table 3:** Physiotherapy intervention N/A: Not applicable, ROM: range of Motion, LSVT: Lee Silverman Voice Treatment

Goals	Intervention	Dosage
Patient Education	A patient's condition, the value of, and the benefits of, physiotherapy are all explained to them. Treatment in improving their health condition, avoiding complications, and increasing her ability to roll, sit, walk, squat, and perform other daily activities with minimal or total independence.	N/A
To improve the flexibility of muscles.	Static stretching to the finger flexors, wrist flexors, and ulnar deviators muscles of the left upper limb.	Static stretching: 3 repetitions each with 30 sec hold, 1 set.
To improve bed mobility	The transition from supine to side lying to sitting was taught.	Every 2 hours
To improve ROM of the upper and lower limbs.	Active exercises for all joints of upper and lower limbs.	1 set. Each set made up of 10 repetitions. Figure 3 shows patient doing active flexion exercise of shoulder joint.
To enhance the strength of upper and lower extremity muscles	Due to shoulder pain, strengthening exercises were delayed for a few days; a half-liter bottle was used as weight for the elbow. Dynamic quads were given for quadriceps strengthening.	1 set. Each set made up of 10 repetitions. Figure 4 shows patient doing dynamic strengthening of quadriceps muscle.
To enhance balance	Progression from standing with feet apart and minimal support to independent with feet closed together, tandem standing.	This exercise was progressed over a period of days, and each position was held for at least 1 minute at the start and then progressed further.
To improve limb movement amplitude	LSVT BIG: Task 1 (maximum sustained movement): Uninterrupted Big “stretch” side to side Uninterrupted Big “stretch” floor to ceiling Task 2 (directional or repetitive movements): Forward, sideways, and backward big step, forward, etc. Task 3 (Functional component movements): Sit to stand	Four consecutive days per week. Started with 8 repetitions of each exercise and progressed to 16. Each activity in Task 1 has a hold of 10 sec for each repetition.
To improve gait	Spot marching progressed to hallway walking and training within parallel bars with auditory cueing.	Twice a day. Figure 5 shows patient doing spot marching with maximal assistance.
To ensure good ventilation	Deep breathing exercises and pursed lip breathing exercises.	10 repetitions, 2 sets of each exercise

Follow-up and outcome measures

After discharge, the patient came to the Neuro Rehabilitation unit for a follow-up for three weeks for rehabilitation. At the end of her treatment, the assessment was done again, and the patient reported improved standing balance and gait and decreased fall risk. The tone of the finger flexors and wrist ulnar deviators was slightly reduced compared to day one. The pre-and post-treatment outcomes are given in Table 4.

**Table 4:** Pre- and post-treatment outcomes FIM: Functional independence measure

	Pre-treatment	Post-treatment
Sitting	Mild support needed	Sits independently
Standing	Moderate assistance needed	Stands with minimal assistance
Walking	Moderate assistance needed	Minimal assistance needed
Berg Balance Scale Score	4	19
FIM	62	84

## Discussion

PD is a neurodegenerative condition that originates from the death of dopamine-producing neurons, eventually leading to decreased neurotransmitter called dopamine. The four basic and important clinical features are bradykinesia, rigidity, resting tremors, and postural instability. The physiotherapeutic approach focuses on treating these four and various motor dysfunctions associated with PD. One study suggested that physiotherapy improved patient’s treatment outcomes compared to the placebo or no treatment group. It also indicated no significant difference in the effects of different interventions. Similarly, we used interventions like stretching, balance, and gait training and found a positive impact on patients [16].

The issue of festinating gait was tackled with gait training with auditory cueing, which included forward-backward walking, change in directions, turning, changing speed, etc. The patient showed improved gait at the end of three weeks. Research conducted by Fietzek et al. showed that patients receiving gait training with audio, visual, and tactile cueing, alone or in combination, showed improved gait and decreased freezing episodes when set side by side with the control group. However, the paper showed no significant improvement in fall prevention [17].

Postural instability is one of the characteristic symptoms of this condition. This led to gait impairment, balance issues, and frequent falls. A recent paper by Youm et al. suggested that using strengthening and stretching exercises of the trunk over three months improved posture balance and decreased falls in this patient [18].

There is improved motor function in PD patients treated with LSVT- BIG. Ebersbach et al. conducted a study to assess and compare short-duration training for amplitude orientation with LSVT-Big and concluded that both equally enhanced motor function [19]. In another research conducted by Schaible et al., he compared the effects of LSVT-BIG and conventional therapy on motor and non-motor symptoms in PD patients. He found that both strategies had a beneficial impact [20].

## Conclusions

As there is a rise in the prevalence of PD worldwide, conducting more thorough and insightful studies on various treatment strategies becomes paramount. This study presents a case of a 62-year-old female previously diagnosed with stage 5 PD, presented with involuntary movements, severe balance impairments, gait impairments, and other motor dysfunctions. She was treated for three weeks with conventional physiotherapy, and LSVT showed improved treatment outcomes.
